# Demographic and Clinical Characteristics of Mpox in Persons Who Had Previously Received 1 Dose of JYNNEOS Vaccine and in Unvaccinated Persons — 29 U.S. Jurisdictions, May 22–September 3, 2022

**DOI:** 10.15585/mmwr.mm715152a2

**Published:** 2022-12-30

**Authors:** Jennifer L. Farrar, Nathaniel M. Lewis, Kennedy Houck, Michelle Canning, Amy Fothergill, Amanda B. Payne, Adam L. Cohen, Joshua Vance, Bridget Brassil, Erin Youngkin, Bailey Glenn, Anil Mangla, Nikki Kupferman, Katharine Saunders, Cristina Meza, Dawn Nims, Susan Soliva, Brandon Blouse, Tiffany Henderson, Emily Banerjee, Brooklyn White, Rachael Birn, Anna M. Stadelman, Meaghan Abrego, Meagan McLafferty, Michael G. Eberhart, Michael Pietrowski, Sandra Miranda De León, Emma Creegan, Abdoulaye Diedhiou, Caleb Wiedeman, Jade Murray-Thompson, Elizabeth McCarty, Jessica Marcinkevage, Anna Kocharian, Elizabeth A. Torrone, Logan C. Ray, Daniel C. Payne, Mpox Cases in Vaccinated Persons Team, Mpox Cases in Vaccinated Persons Team, Matthew Cole, Lauren Roper, Hazel Shah, Louise McNitt, Stephanie Gretsch, Melissa Pike, Patricia Firmender, Will Still, Jamie Ahlers, Aman Punwani, Komal Patel, Nam-Kyu Cho, Marcia Pearlowitz, Petra Schubert, Ryan Malosh, Sydney Kuramoto, Matthew Donahue, Miranda Durham, Charlotte DelBarba, Kelly Cogswell, Julie Miedlar, Dana Perella, Julian D. Cordero Calderon, Taidy Perez, Jacqueline Logan, Abigail Collingwood, Naihlah Smith, Rachel Klos

**Affiliations:** ^1^CDC Mpox Emergency Response Team; ^2^Epidemic Intelligence Service, CDC; ^3^California Department of Public Health; ^4^Immunization Services Division, National Center for Immunization and Respiratory Diseases, CDC; ^5^Chicago Department of Public Health, Chicago, Illinois; ^6^Colorado Department of Public Health and Environment; ^7^Connecticut Department of Public Health; ^8^Council of State and Territorial Epidemiologists, Atlanta, Georgia; ^9^District of Columbia Department of Health; ^10^Delaware Department of Health and Social Services; ^11^Florida Department of Health; ^12^Georgia Department of Public Health; ^13^Illinois Department of Public Health; ^14^Massachusetts Department of Public Health; ^15^Maryland Department of Health; ^16^Michigan Department of Health and Human Services; ^17^Minnesota Department of Health; ^18^Missouri Department of Health and Senior Services; ^19^Nebraska Department of Health and Human Services; ^20^New Mexico Department of Health; ^21^New York State Department of Health; ^22^Oregon Health Authority, Public Health Division; ^23^Pennsylvania Department of Health; ^24^City of Philadelphia Department of Public Health, Philadelphia, Pennsylvania; ^25^Puerto Rico Department of Health; ^26^Rhode Island Department of Health; ^27^South Carolina Department of Health and Environmental Control; ^28^Tennessee Department of Health; ^29^Utah Department of Health and Human Services; ^30^Virginia Department of Health; ^31^Washington State Department of Health; ^32^Wisconsin Department of Health Services.; CDC 2022 Multinational Monkeypox Vaccine Effectiveness Team; CDC 2022 Multinational Monkeypox Informatics Team; CDC 2022 Multinational Monkeypox Vaccine Data Team; California Department of Public Health; Chicago Department of Public Health; Colorado Department of Public Health and Environment; Connecticut Department of Public Health; District of Columbia Department of Health; Delaware Health and Social Services; Florida Department of Health; Georgia Department of Public Health; Illinois Department of Public Health; Maryland Department of Health; Massachusetts Department of Public Health; Michigan Department of Health and Human Services; Minnesota Department of Health; Nebraska Department of Health and Human Services; New Mexico Department of Health; New York State Department of Health; Oregon Health Authority, Public Health Division; Pennsylvania Department of Health; City of Philadelphia Department of Public Health; Puerto Rico Department of Health; Monkeypox Team at the Center for Acute Infectious Disease Epidemiology at Rhode Island Department of Health; South Carolina Department of Health and Environmental Control; Tennessee Department of Health; Utah Department of Health and Human Services; Washington State Department of Health; Wisconsin Department of Health Services.

As of November 14, 2022, monkeypox (mpox) cases had been reported from more than 110 countries, including 29,133 cases in the United States.* Among U.S. cases to date, 95% have occurred among males (*1*). After the first confirmed U.S. mpox case on May 17, 2022, limited supplies of JYNNEOS vaccine (Modified Vaccinia Ankara vaccine, Bavarian Nordic) were made available to jurisdictions for persons exposed to mpox. JYNNEOS vaccine was approved by the Food and Drug Administration (FDA) in 2019 as a 2-dose series (0.5 mL per dose, administered subcutaneously) to prevent smallpox and mpox disease.^†^ On August 9, 2022, FDA issued an emergency use authorization to allow administration of JYNNEOS vaccine by intradermal injection (0.1 mL per dose) (*2*). A previous report on U.S. mpox cases during July 31–September 3, 2022, suggested that 1 dose of vaccine offers some protection against mpox (*3*). This report describes demographic and clinical characteristics of cases occurring ≥14 days after receipt of 1 dose of JYNNEOS vaccine and compares them with characteristics of cases among unvaccinated persons with mpox and with the vaccine-eligible vaccinated population in participating jurisdictions. During May 22–September 3, 2022, among 14,504 mpox cases reported from 29 participating U.S. jurisdictions,^§^ 6,605 (45.5%) had available vaccination information and were included in the analysis. Among included cases, 276 (4.2%) were among persons who had received 1 dose of vaccine ≥14 days before illness onset. Mpox cases that occurred in these vaccinated persons were associated with lower percentage of hospitalization (2.1% versus 7.5%), fever, headache, malaise, myalgia, and chills, compared with cases in unvaccinated persons. Although 1 dose of JYNNEOS vaccine offers some protection from disease, mpox infection can occur after receipt of 1 dose, and the duration of protection conferred by 1 dose is unknown. Providers and public health officials should therefore encourage persons at risk for acquiring mpox to complete the 2-dose vaccination series and provide guidance and education regarding nonvaccine-related prevention strategies (*4*).

Probable and confirmed mpox cases^¶^ among persons with illness onset during May 22–September 3, 2022, in the 29 jurisdictions were eligible for inclusion. Persons who had received 1 dose of JYNNEOS vaccine ≥14 days before illness onset were considered vaccinated for the purposes of this study**; those who had not received 1 vaccine dose during the current outbreak or who reported illness onset before receipt of their first vaccine dose were considered unvaccinated. Cases were excluded if 1) no vaccination date or vaccination status was available, 2) receipt of vaccine occurred before May 2022, or 3) illness onset occurred ≤13 days after receipt of 1 vaccine dose.

Participating jurisdictions collected data using a standardized data collection form^††^ including self-reported demographic characteristics, vaccination history, medical history, and possible exposures. Participating jurisdictions linked vaccination data from immunization registries when available or by self-report during case investigation and transmitted the linked data to CDC.

Demographic characteristics of persons with mpox who had received 1 vaccine dose (i.e., were vaccinated) were compared with those of unvaccinated persons with mpox. In addition, characteristics of persons with mpox who were vaccinated were compared with those of all vaccine-eligible persons who were vaccinated, irrespective of case status; these data were obtained through jurisdictional immunization registries reporting first doses administered. Comparisons were made using Pearson’s chi-square test, Fisher’s exact test, or the Wilcoxon rank-sum test as appropriate. P-values <0.05 were considered statistically significant.

To assess differences in illness among vaccinated and unvaccinated persons with mpox, clinical characteristics were compared between these two groups among persons with data reported for one or more clinical symptoms. Missing individual symptom data were imputed as “no” when there was evidence that the reporting jurisdiction collected symptom data in a “check-all-that-apply” format.^§§^ Odds ratios and 95% CIs were calculated to compare clinical characteristics of vaccinated and unvaccinated mpox patients. Persons with missing data for relevant variables of interest were excluded from individual analyses.

Because JYNNEOS vaccine was administered for postexposure prophylaxis at the beginning of the outbreak, and because the mpox incubation period can be as long as 21 days, a sensitivity analysis including only persons with mpox who received 1 vaccine dose ≥22 days before illness onset as the vaccinated group was conducted. SAS (version 9.4; SAS Institute) and R (version 4.0.3; R Foundation) were used to conduct all analyses. This activity was reviewed by CDC and was conducted consistent with applicable federal law and CDC policy.^¶¶^

During May 22, 2022–September 3, 2022, a total of 14,504 mpox cases were reported in the 29 included jurisdictions. Among the 6,605 (45.5%) persons with mpox who had available information and who were included in the analysis,*** 6,329 (95.8%) were unvaccinated, and 276 (4.2%) had illness onset ≥14 days after receiving 1 vaccine dose (Table 1). Among vaccinated patients, the median interval from vaccination to illness onset was 23 days (IQR = 17.5–30 days). The age distribution differed for vaccinated mpox patients compared with the vaccine-eligible population identified through immunization registries (p<0.001), but not compared with unvaccinated mpox patients (p = 0.07); 91.2% of vaccinated mpox patients were aged 18–49 years. Overall, 68.2% of vaccinated mpox patients and 49.9% of unvaccinated mpox patients reported White race, whereas 12.4% and 30.9% of vaccinated and unvaccinated patients, respectively, reported Black or African American race (p<0.001). Among 345,220 vaccine-eligible persons in the population who had received 1 dose of JYNNEOS vaccine, a significantly smaller percentage identified as White (46.7%) compared with vaccinated mpox patients (62.8%; p = 0.02). Overall, 259 (98.1%) vaccinated and 5,710 (96.1%) unvaccinated mpox cases occurred in persons identifying as male.

**TABLE 1:** Characteristics of mpox patients, by vaccination status (N = 6,605) and of recipients of 1 dose of JYNNEOS vaccine (N = 345,220) — 29 U.S. jurisdictions,* May 22–September 3, 2022

Characteristic	Mpox patient vaccination status, n/N (%)^†^		p-value**	Recipients of 1 vaccine dose, n/N (column %) (N = 345,220)^††^	p-value**^,§§^
	Vaccinated^§^ (n = 276)	Unvaccinated^¶^ (n = 6,329)			
**Age group, yrs**					
Mean (median)	36.9 (36.0)	35.3 (34.0)	<0.01	NA	NA
18–29	57/275 (20.7)	1,755/6,282 (27.9)	0.07	72,998/345,215 (21.1)	<0.001
30–39	134/275 (48.7)	2,714/6,282 (43.2)		113,503/345,215 (32.9)	
40–49	60/275 (21.8)	1,266/6,282 (20.2)		64,481/345,215 (18.7)	
≥50	24/275 (8.7)	547/6,282 (8.7)		93,768/345,215 (27.2)	
Missing	1	47		5	
**Race**					
American Indian or Alaska Native	2/266 (0.8)	43/6,140 (0.7)	<0.001	975/345,220 (0.2)	0.02
Asian	17/266 (6.4)	196/6,140 (3.2)		23,598/345,220 (6.8)	
Black or African American	33/266 (12.4)	1,901/6,140 (30.9)		37,325/345,220 (10.8)	
Native Hawaiian or other Pacific Islander	1/266 (0.4)	19/6,140 (0.3)		783/345,220 (0.2)	
White	167/266 (62.8)	3,054/6,140 (49.9)		161,203/345,220 (46.7)	
Multiracial or other	8/266 (3.0)	275/6,140 (4.5)		15,247/345,220 (4.4)	
Unknown	38/266 (14.3)	652/6,140 (10.6)		34,948/345,220 (10.1)	
Missing	10	189		0	
**Ethnicity**					
Hispanic or Latino	64/269 (23.8)	1,880/6,159 (30.5)	0.06	71,141/345,220 (20.6)	0.02
Non-Hispanic	177/269 (65.8)	3,929/6,159 (63.8)		274,079/345,220 (79.4)	
Unknown	28/269 (10.4)	350/6,159 (5.7)		0	
Missing	7	170		0	
**Sex at birth**					
Female	0	137/5,956 (2.3)	0.02	24,306/345,220 (7.0)	<0.001
Male	216/257 (84.1)	5,408/5,956 (90.8)		315,940/345,220 (91.5)	
Unknown	41/257 (15.4)	411/5,956 (6.9)		4,974/345,220 (1.4)	
Missing	19	373		0	
**Gender**					
Female	1/264 (0.3)	127/5,939 (2.2)	0.20	NA	NA
Male	259/264 (98.1)	5,710/5,939 (96.1)		NA	
Transgender female	0	37/5,939 (0.6)		NA	
Transgender male	1/264 (0.3)	18/5,939 (0.3)		NA	
Another gender identity	3/264 (1.1)	47/5,939 (0.8)		NA	
Missing	12	390		NA	

**Abbreviations**: mpox = monkeypox; NA = not applicable.

* California; Chicago, Illinois; Colorado; Connecticut; Delaware; District of Columbia; Florida; Georgia; Illinois; Massachusetts; Maryland; Michigan; Minnesota; Missouri; Montana; Nebraska; New Mexico; New York (not including New York City); Oregon; Pennsylvania; Philadelphia, Pennsylvania; Puerto Rico; Rhode Island; South Carolina; Tennessee; Utah; Virginia; Washington; and Wisconsin.

^†^ Percentages were calculated using cases with available data as the denominator.

^§^ Cases of probable and confirmed mpox in persons with illness onset ≥14 days after receipt of 1 vaccine dose.

^¶^ Cases of probable and confirmed mpox in persons with illness onset before the date of vaccination or who did not report receipt of vaccine.

** Medians were compared using a Wilcoxon rank sum test. Proportions were compared using Pearson’s chi-square test or Fisher’s exact test.

^††^ Includes all persons within the vaccine-eligible population who received 1 vaccine dose, including vaccinated mpox patients.

^§§ ^Comparison between recipients of 1 vaccine dose in the vaccine-eligible population and vaccinated mpox patients.

Information on at least one clinical finding was available for 202 (73.2%) of 276 vaccinated persons with mpox and 5,326 (84.2%) of 6,329 unvaccinated mpox patients. Among those who were vaccinated, the most commonly reported signs and symptoms were rash (96.5%), pruritis (33.5%), and enlarged lymph nodes (31.6%) (Table 2). Among unvaccinated persons, the most common signs and symptoms were rash (97.3%), fever (46.5%), and malaise (43.0%). The odds of fever, headache, malaise, abdominal pain, vomiting or nausea, myalgia, and chills were significantly lower among vaccinated than among unvaccinated patients (Figure). Odds of rectal signs and symptoms (e.g., proctitis, rectal bleeding, tenesmus, and rectal pain) were similar among vaccinated and unvaccinated mpox patients.

**TABLE 2:** Clinical characteristics of mpox patients, by vaccination status* — 29 U.S. jurisdictions,^†^ May 22–September 3, 2022

Characteristic	Mpox patient vaccination status, n/N (%)		Odds ratio (95% CI)
	Vaccinated^§^ (n = 202)	Unvaccinated^¶^ (n = 5,326)	
**HIV status**			
Positive	19/78 (24.4)	1,074/2,585 (41.6)	Not calculated
Negative	46/78 (58.9)	1,153/2,585 (44.5)	
Unknown or missing	137	3,099	
**Signs and symptoms**			
Abdominal pain	5/190 (2.6)	420/5,069 (8.3)	0.29 (0.12–0.72)
Chills	23/193 (11.9)	2,015/5,207 (38.7)	0.21 (0.14–0.33)
Conjunctivitis	2/65 (3.1)	148/2,703 (5.5)	0.57 (0.14–2.36)
Enlarged lymph nodes	62/196 (31.6)	1,841/5,237 (35.2)	0.84 (0.62–1.14)
Fever	55/198 (27.8)	2,451/5,266 (46.5)	0.44 (0.32–0.60)
Headache	39/195(20.0)	1,908/5,217 (36.6)	0.43 (0.30–0.61)
Malaise	47/192 (24.5)	2,240/5,204 (43.0)	0.43 (0.31–0.60)
Myalgia	39/194 (20.1)	1,696/5,212 (32.5)	0.52 (0.36–0.74)
Proctitis	11/187 (5.9)	360/4,983 (7.2)	0.81 (0.44–1.50)
Pruritis	65/194 (33.5)	1,840/5,193 (35.4)	0.93 (0.68–1.26)
Pus in stool	12/191 (6.3)	558/5,169 (10.8)	0.55 (0.30–0.99)
Rectal bleeding	17/193 (8.8)	684/5,182 (13.2)	0.63 (0.38–1.04)
Rectal pain	38/194 (19.6)	1,255/5,211 (24.1)	0.77 (0.53–1.10)
Tenesmus	16/190 (8.4)	573/5,164 (11.1)	0.74 (0.44–1.24)
Vomiting or nausea	6/199 (3.0)	494/5,263 (9.4)	0.31 (0.13–0.69)
**Rash**			
Presence of rash	195/202 (96.5)	5,173/5,318 (97.3)	0.73 (0.34–1.58)
**Rash location**			
Arms	20/160 (12.5)	1,860/4,140 (44.9)**	0.18 (0.11–0.28)
Face	26/160 (16.3)	1,386/4,140 (33.5)**	0.39 (0.25–0.59)
Genitals	88/160 (55.0)	1,933/4,140 (46.7)**	1.40 (1.02–1.92)
Head	27/160 (16.9)	1,376/4,140 (33.2)**	0.41 (0.27–0.62)
Legs	19/160 (11.9)	1,628/4,140 (39.3)**	0.21 (0.13–0.34)
Mouth, lips, or oral mucosa	7/160 (4.4)	400/4,140 (9.7)**	0.43 (0.20–0.92)
Neck	5/160 (3.13)	545/4,140 (13.2)**	0.21 (0.09–0.52)
Palms of hands	6/160 (3.8)	806/4,140 (19.5)**	0.16 (0.07–0.37)
Perianal	42/160 (26.3)	1,212/4,140 (29.3)	0.86 (0.60–1.23)
Soles of feet	1/160 (0.6)	445/4,140 (10.8)**	0.05 (0.01–0.37)
Trunk	18/160 (11.3)	1,521/4140 (36.7)**	0.22 (0.13–0.36)
Other locations	68/160 (42.5)	1,574/4,140 (38.0)	1.21 (0.88–1.66)
**Number of rash locations reported**			
Median (IQR)**	2.0 (1.0–3.0)	3.0 (2.0–5.0)	<0.001^††^
**Severity**			
Hospitalization related to mpox	2/95 (2.1)	237/3,142 (7.5)	0.27 (0.06–1.09)

**Abbreviation:** mpox = monkeypox.

* Persons with complete data for one or more clinical symptom were included in analysis; for vaccinated persons 73.2% were included (202/276), and for unvaccinated persons 84.2% were included (5,326/6,329).

^†^ California; Chicago, Illinois; Colorado; Connecticut; Delaware; District of Columbia; Florida; Georgia; Illinois; Massachusetts; Maryland; Michigan; Minnesota; Missouri; Montana; Nebraska; New Mexico; New York (not including New York City); Oregon; Pennsylvania; Philadelphia, Pennsylvania; Puerto Rico; Rhode Island; South Carolina; Tennessee; Utah; Virginia; Washington; and Wisconsin.

^§^ Cases of probable and confirmed mpox in persons with illness onset ≥14 days after receipt of 1 vaccine dose (202/276).

^¶^ Cases of probable and confirmed mpox in persons with illness onset before the date of vaccination or who did not report receipt of vaccine (5,326/6,329).

** Median rash locations reported from 160 vaccinated and 4,140 unvaccinated mpox patients.

^††^ Medians compared using Wilcoxon rank sum test.

**FIGURE:**
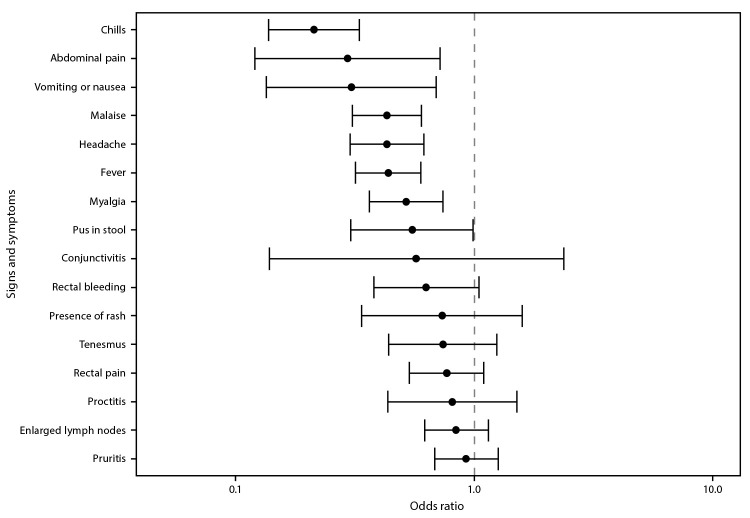
Odds* of signs and symptoms present among persons with mpox who received 1 dose of JYNNEOS vaccine compared with those in unvaccinated persons with mpox — 29 U.S. jurisdictions,^†^ May 22–September 3, 2022 * With 95% CIs indicated by error bars. **^†^** California; Chicago, Illinois; Colorado; Connecticut; Delaware; District of Columbia; Florida; Georgia; Illinois; Massachusetts; Maryland; Michigan; Minnesota; Missouri; Montana; Nebraska; New Mexico; New York (not including New York City); Oregon; Pennsylvania; Philadelphia, Pennsylvania; Puerto Rico; Rhode Island; South Carolina; Tennessee; Utah; Virginia; Washington; and Wisconsin.

Among both vaccinated and unvaccinated patients, the genital area was the rash location most commonly reported (55.0% of vaccinated patients and 46.7% of unvaccinated patients) (Table 2). The odds of reporting rash in all other locations except the perianal area were significantly lower among vaccinated than among unvaccinated patients. The median number of rash locations reported by vaccinated patients (two) was significantly lower than that reported by unvaccinated patients (three) (p<0.001). Among 129 persons with mpox who received 1 vaccine dose ≥22 days before illness onset, demographic and clinical findings were not different from those in persons who had received vaccine ≥14 days earlier.

Among 3,142 unvaccinated persons with mpox, 237 (7.5%) were hospitalized compared with two (2.1%) of 95 vaccinated mpox patients (odds ratio = 0.27; 95% CI = 0.06–1.09). No deaths were reported in either group.

## Discussion

In this analysis of 276 mpox cases in persons who received 1 dose of JYNNEOS vaccine ≥14 days before illness onset and 6,329 cases in unvaccinated persons during the 2022 U.S. outbreak, vaccinated patients reported signs and symptoms similar to those described earlier in the outbreak (*1*); however, some symptoms were reported less frequently among vaccinated than among unvaccinated mpox patients. In addition, the percentage of vaccinated patients who were hospitalized (2%) was lower than that among unvaccinated patients (8%), and the odds of systemic signs and symptoms, such as fever and chills, were lower among vaccinated patients. These findings indicate that 1 dose of the JYNNEOS vaccine might attenuate the severity of mpox illness in persons who are infected after vaccination.

The frequent presentation of rash in the genital and perianal areas among both vaccinated and unvaccinated mpox patients suggests that sexual transmission in this population was a common mechanism of transmission. The fewer number of reported rash locations among vaccinated patients suggests possible prevention of spread of rash from site of inoculation among even partially vaccinated persons.

The predominance of White persons among vaccinated mpox patients compared with unvaccinated patients reflects the ongoing racial and ethnic disparities in receipt of the JYNNEOS vaccine nationwide (*5*,*6*) and could indicate differential access to or acceptance of the vaccine. Disparities in access to health care and additional treatment options could also have played a role in decreasing the severity of illness in vaccinated White persons.

The findings in this report are subject to at least four limitations. First, 47% of cases were excluded because of missing vaccination information; therefore, results might not be generalizable to all persons with mpox in the United States. Second, persons with mpox who received vaccine outside of their respective jurisdictions of residence might not have had documentation of vaccine receipt, which could lead to potential undercounting of cases among vaccinated persons (*7*), although many jurisdictions did report sharing vaccination data with one another (Sarah Gillani, District of Columbia Department of Health, personal communication, September 2022). Third, self-reported doses were included to optimize ascertainment of vaccination status, but self-report is less accurate than documented vaccine receipt and could result in misclassification of vaccination status. Finally, clinical data were not available for all cases. In particular, HIV status was unknown or missing for two thirds of vaccinated and more than one half of unvaccinated patients. Because HIV infection might affect the trajectory and illness severity, future studies with additional data for real-world use of JYNNEOS vaccine against clinical outcomes, including data on HIV history, are needed.

The more limited distribution of rash and reduced severity of illness among persons who had mpox after receiving 1 JYNNEOS vaccine dose supports the potential benefit of vaccination on attenuation of disease. Although infection ≥14 days after receipt of 1 JYNNEOS vaccine dose is infrequent, the occurrence of such cases and the unknown duration of protection conferred by 1 vaccine dose highlights the need for providers and public health officials to encourage completion of the 2-dose vaccination series among persons at risk and continue to provide guidance and education regarding nonvaccine-related prevention strategies (*4*) until optimal immune protection from the second dose is achieved.

**Summary**

**What is already known about this topic?**

Evidence suggests that 1 dose of JYNNEOS vaccine offers some protection against monkeypox (mpox).

**What is added by this report?**

Analysis of mpox infections among unvaccinated persons and those who had received 1 JYNNEOS vaccine dose ≥14 days before illness onset found that the odds of fever, headache, malaise, myalgia, and chills were significantly lower among vaccinated patients than among unvaccinated patients. Overall, 2% of vaccinated persons with mpox and 8% of unvaccinated patients were hospitalized.

**What are the implications for public health practice?**

One dose of JYNNEOS vaccine might attenuate the severity of illness and reduce hospitalization in persons who become infected after vaccination; however, to optimize protection, all eligible persons are recommended to complete the 2-dose vaccination series.

## Footnotes

*
https://www.cdc.gov/poxvirus/monkeypox/response/2022/world-map.html

†
https://www.fda.gov/media/131078/download

§ California; Chicago, Illinois; Colorado; Connecticut; Delaware; District of Columbia; Florida; Georgia; Illinois; Massachusetts; Maryland; Michigan; Minnesota; Missouri; Montana; Nebraska; New Mexico; New York (not including New York City); Oregon; Pennsylvania; Philadelphia, Pennsylvania; Puerto Rico; Rhode Island; South Carolina; Tennessee; Utah; Virginia; Washington; and Wisconsin.

¶
https://www.cdc.gov/poxvirus/monkeypox/clinicians/case-definition.html

** For this analysis, persons were considered vaccinated ≥14 days after receipt of 1 dose of JYNNEOS vaccine, based on previous immunogenicity studies showing an immunologic response 14 days after vaccination.

††
https://www.cdc.gov/poxvirus/monkeypox/pdf/scrf-short-form.pdf

§§ Jurisdictions were considered to have collected symptom variables in a “check-all-that-apply” manner if a case patient had a “yes” response for one or more clinical symptoms, but all other symptoms were reported as missing.

¶¶ 5 C.F.R. part 46, 21 C.F.R. part 56; 42 U.S.C. Sect. 241(d); 5 U.S.C. Sect. 552a; 44 U.S.C. Sect. 3501 et seq.

*** A total of 6,856 (47.3%) cases were excluded because information on vaccination status or date of vaccination was missing, 106 (0.7%) had a vaccine date before May 2022 and were excluded, and 927 (6.4%) were excluded because they had been vaccinated <14 days from illness onset.
